# CF_4_ Plasma‐Generated LiF‐Li_2_C_2_ Artificial Layers for Dendrite‐Free Lithium‐Metal Anodes

**DOI:** 10.1002/advs.202201147

**Published:** 2022-05-26

**Authors:** Shengling Cao, Xin He, Lanlan Nie, Jianwei Hu, Manlin Chen, Yu Han, Kangli Wang, Kai Jiang, Min Zhou

**Affiliations:** ^1^ State Key Laboratory of Advanced Electromagnetic Engineering and Technology School of Electrical and Electronic Engineering Huazhong University of Science and Technology Wuhan 430074 China; ^2^ State Key Laboratory of Materials Processing and Die & Mould Technology School of Materials Science and Engineering Huazhong University of Science and Technology Wuhan 430074 China

**Keywords:** CF_4_ plasma, dendrite‐free, LiF‐Li_2_C_2_ artificial layer, lithium metal anodes

## Abstract

Lithium metal anodes have long been considered as “holy grail” in the field of energy storage batteries, but dendrite growth and large volume changes hinder their practical applications. Herein, a facile and eco‐friendly CF_4_ plasma treatment is employed for the surface modification of Li anodes, and an artificial layer consisting of LiF and Li_2_C_2_ is fabricated for the first time. Experimental results and theoretical calculations reveal that the high adsorption energy of LiF and low Li^+^ diffusion barriers in Li_2_C_2_ induce uniform nucleation and planar growth of Li, guaranteeing a stable and dendrite‐free Li structure during the repeated plating/stripping process of cycling. Symmetric cells using CF_4_ plasma‐treated Li operate stably for more than 6500 h (at 2 mA cm^−2^ and 1 mAh cm^−2^) or 950 h (at 1 mA cm^−2^ and 10 mAh cm^−2^). When paired with a LiFePO_4_ cathode, full batteries deliver a high reversible capacity of 136 mAh g^−1^ (at 1 C) with considerable cycling stability (97.2% capacity retention over 200 cycles) and rate performance (116 mAh g^−1^ up to 5 C). This powerful application of plasma technology toward novel LiF‐Li_2_C_2_ artificial layers provide new routes for constructing environment‐friendly and high‐performance energy storage devices.

## Graphical abstract

An artificial layer consisting of LiF and Li_2_C_2_ is fabricated by a facile and eco‐friendly CF_4_ plasma treatment for the first time. The symmetric cells using the CF_4_ plasma‐treated Li can operate stably for more than 6500 h with an overpotential of 50 mV.

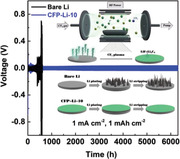

## 1 Introduction

Lithium metal is considered as the ideal anode material for high‐energy‐density batteries, owing to its ultrahigh theoretical capacity (3860 mAh g^−1^) and low redox potential (3.04 V with respect to a standard hydrogen electrode).^[^ ^1^ ^]^ However, safety issues and shortened lifespans significantly impede the practical application of lithium metal batteries (LMBs).^[^ ^2^ ^]^ These issues stem from the high reactivity of Li, which causes continuous consumption of the liquid electrolyte to form a solid electrolyte interface (SEI).^[^ ^3^ ^]^ In turn, the inhomogeneous SEI layer shows a low interfacial energy with Li,^[^ ^4^ ^]^ leading to non‐uniform dendritic growth of Li.^[^ ^5^ ^]^ Uncontrollable growth of Li dendrites can easily rupture the formed SEI layer,^[^ ^6^ ^]^ resulting in poor coulombic efficiencies.^[^ ^7^ ^]^ Moreover, the accumulation of dendritic Li can eventually penetrate separators and cause hazards.^[^ ^8^ ^]^ Last, large volume changes during repeated Li plating/stripping inevitably disrupt internal contact^[^ ^9^ ^]^ and limit the energy and power densities of LMBs.^[^ ^10^ ^]^

An efficient strategy for solving these issues is the engineering of a stable artificial SEI layer with sufficient strength and high interfacial energy.^[^ ^11^ ^]^ One of the key components in the SEI layer,^[^ ^12^ ^]^ LiF, has attracted increasing attention due to its desirable interfacial properties, high Young's modulus,^[^ ^13^ ^]^ and large interfacial energy versus Li metal (73.28 meV Å ^−2^).^[^ ^14^ ^]^ LiF‐rich SEI layers, with their relatively low diffusion barrier for Li^+^, can also effectively induce homogeneous nucleation and planar growth of lithium.^[^ ^15^ ^]^ In addition to LiF, Li_2_C_2_ has also been identified as a substantial component of the SEI that forms at potentials below 0 V (vs Li/Li^+^).^[^ ^16^ ^]^ Recent research has shown that Li_2_C_2_ demonstrates excellent potential for constructing dendrite‐free Li anodes with low polarization.^[^ ^17^ ^]^ Based on these considerations, the fabrication of an artificial SEI layer simultaneously containing LiF and Li_2_C_2_ should be an effective strategy for constructing dendrite‐free and cycling‐stable LMBs.

To date, the decoration of lithium metal with Li_2_C_2_ has seldom been reported,^[^ ^16^ ^]^ as investigation of the structure and properties of Li_2_C_2_ is in its infancy. In contrast, tremendous efforts have been devoted to constructing LiF‐rich SEI layers.^[^ ^18^ ^]^ One major strategy toward this aim is introducing F‐containing electrolyte additives (e.g., HF,^[^ ^19^ ^]^ LiF,^[^ ^20^ ^]^ and FEC^[^ ^21^ ^]^) for the in situ formation of F‐rich SEI films. The F‐containing additives were primarily decomposed on the surface of the anode owing to their lower LUMO energy levels.^[^ ^22^ ^]^ F‐rich SEI layers possess self‐healing abilities, and can effectively protect the anode.^[^ ^23^ ^]^ However, limited amounts of functional additives are consumed during the repeated charging‐discharging process;^[^ ^24^ ^]^ this cannot guarantee long‐term cycling stability. Other schemes for ex situ fabrication of LiF layers involve directly coating LiF on the surface of the anode. However, such fabrication methods, including atomic layer deposition (ALD),^[^ ^25^ ^]^ physical vapor deposition (PVD),^[^ ^26^ ^]^ and directly coated with LiF,^[^ ^27^ ^]^ are time consuming.^[^ ^27^, ^28^ ^]^ In addition, high temperatures are required for the reaction of certain fluorides;^[^ ^29^ ^]^ this introduces impurities on the surface of the lithium metal and causes unexpected side reactions.^[^ ^30^ ^]^ Therefore, there is an urgent need for a simple and scalable strategy for the coating of Li surfaces at the atomic level with thin, uniform LiF films.

Plasma, generated by high‐voltage ionization^[^ ^31^ ^]^ comprises highly reactive electrons, ions, and neutral species.^[^ ^32^ ^]^ Plasma reagents can easily form numerous active sites on the material surface through energy exchange,^[^ ^33^ ^]^ allowing reactions with high barriers to proceed under mild conditions within a few minutes.^[^ ^34^ ^]^ Through regulation of feed gas composition and vacuum level, particular species can be modified at the atomic level on material surfaces without the introduction of impurities.^[^ ^35^ ^]^ Moreover, compared to other traditional treatments, the high degree of control, low operating temperatures, and short reaction times present in plasma treatments allow for maximum preservation of bulk properties and low environmental impact.^[^ ^36^ ^]^ Therefore, non‐thermal plasma treatment appears to be a promising approach for high‐quality modification of Li metal anodes.^[^ ^35a^ ^]^

In this study, a CF_4_ plasma was employed as both the fluorine and carbon source for the surface treatment of Li anodes. The CF_4_ precursor provided highly reactive F^*^ and C_2_ species through gas ionization, forming LiF and Li_2_C_2_ on Li metal surfaces via ion bombardment. The resulting thin and uniform artificial layers composed of LiF and Li_2_C_2_ favored uniform nucleation of lithium ions,^[^ ^37^ ^]^ and thus inhibited the growth of lithium dendrites. Benefiting from the high adsorption energy, low diffusion barrier, and strong mechanical strength of the LiF‐Li_2_C_2_ composite layer, symmetric cells using plasma‐treated Li showed optimal electrochemical performance: exhibiting stable cycling behavior over 6500 h and a low voltage hysteresis of 50 mV. When coupled with LiFePO_4_ (LFP) cathodes, full batteries using CF_4_ plasma treated lithium metal anodes delivered a high reversible capacity of 136 mAh g^−1^ with good cycling stability and rate performance. Thus, the use of plasma treatment to generate artificial LiF/Li_2_C_4_ layers shows excellent potential for enabling practical applications of high‐performance LMBs with dendrite‐free Li growth and long‐term cycling stability.

## 2 Results and Discussion

**Scheme 1:**
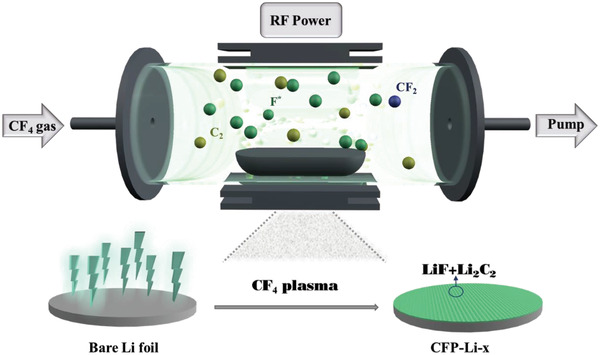
Schematic illustration of the plasma discharge mechanism and the formation of artificial LiF and Li_2_C_2_.

**Figure 1:**
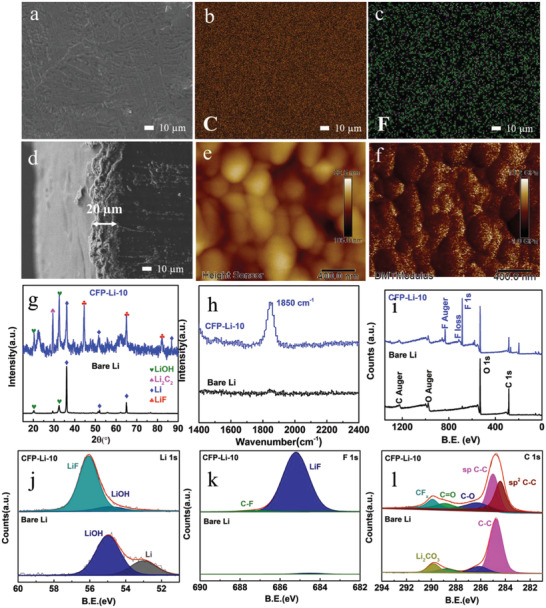
Structural and chemical characterization of CPS‐Li‐10: a) top‐view SEM image, b) carbon EDS elemental map, c) fluorine EDS elemental map, d) cross‐sectional SEM image, e) AFM tomograph, f) Young's modulus map, g) grazing incident X‐ray diffraction (GIXRD), h) Raman spectrum, i) X‐ray photoelectron spectroscopy (XPS) spectra, j) XPS spectra for lithium 1s, k) fluorine 1s, and l) carbon 1s of bare Li and CFP‐Li‐10.

CF_4_ plasma primarily comprises highly active F*, F_2_, CF_2_, and C_2_ groups (Figure S1, Supporting Information). During plasma treatment, high‐energy C‐ and F‐containing species bombard the Li surface and react with Li to form an artificial layer comprising LiF and Li_2_C_2_ (**Scheme** **1**). Herein, to analyze the effect of plasma treatment time, lithium foils were treated with CF_4_ plasma for 5, 10, and 20 min; these are referred to as CFP‐Li‐5, CFP‐Li‐10, and CFP‐Li‐20, respectively. Scanning electron microscopy (SEM) images of the treated foils (**Figure** **1**a; Figure S2b,c, Supporting Information) show that the LiF/Li_2_C_2_ coating was fabricated as a uniform layer with a porous structure. EDS mapping (Figure 1b,c) of CFP‐Li‐10 suggests a uniform distribution of C and F. As the treatment time was extended, the thickness of the artificial layer increased: 2, 18, and 93 µm for 5, 10, and 20 min, respectively (Figure 1d; Figure S2d,e, Supporting Information). Atomic force microscopy (AFM) was further used to study the surface roughness and mechanical strength of the artificial layer in CFP‐Li‐10 (Figure 1e). The Young's modulus (Figure 1f) of CFP‐Li‐10 was calculated to be 4.3 GPa, much higher than that of bare Li foil (2.1 GPa, Figure S3b, Supporting Information). The high mechanical strength of the artificial layer is conjectured to effectively restrain dendritic Li growth and accommodate the volume change during the repeated plating/stripping process.^[^ ^38^ ^]^

To obtain a precise structural characterization of the Li surface, grazing incidence X‐ray diffraction (GIXRD) with a detection depth in the micron range was conducted. As shown in Figure 1g, peaks located at 45.0°, 65.5°, and 83.0° were seen for plasma‐treated Li (CFP‐Li‐10); these correspond to the formation of LiF (PDF#45‐1460). In addition, the strong diffraction peak at 29.5° can be indexed to the (110) plane of orthorhombic Li_2_C_2_ (PDF#21‐0484). Notably, diffraction peaks for LiOH were observed in the XRD patterns of both pure Li and CFP‐Li‐10, indicating slight surface oxidation of the Li foil during the test. X‐ray photoelectron spectroscopy (XPS) characterization was also performed to further study the surface electronic states present in plasma‐treated Li. As illustrated in Figure 1i, the XPS survey spectra confirmed the existence of Li, F, O, and C on the surface of CFP‐Li‐10, with molar contents of 52.7%, 31.9%, 5.2%, and 10.2%, respectively (after eliminating the carbon signal resulting from the XPS instrument itself). The peaks at 56.1 eV in the high‐resolution spectra for Li 1s (Figure 1j) and 685.2 eV in the F 1s spectra (Figure 1k) confirm the formation of LiF. In the C 1s high‐resolution spectra (Figure 1l), the peak located at a binding energy of 285.0 eV was assigned to the sp‐hybridized carbon in Li_2_C_2_; this is consistent with the GIXRD results. As further confirmation of the formation of Li_2_C_2_, a characteristic peak seen at 1850 cm^−1^ in the Raman spectrum for CFP‐Li‐10 can be attributed to the symmetrical stretching vibration of the C≡C bond (Figure 1h). Based on the above characterizations, the surface layer of CFP‐Li‐10 can be concluded to be composed of LiF and Li_2_C_2_.

**Figure 2:**
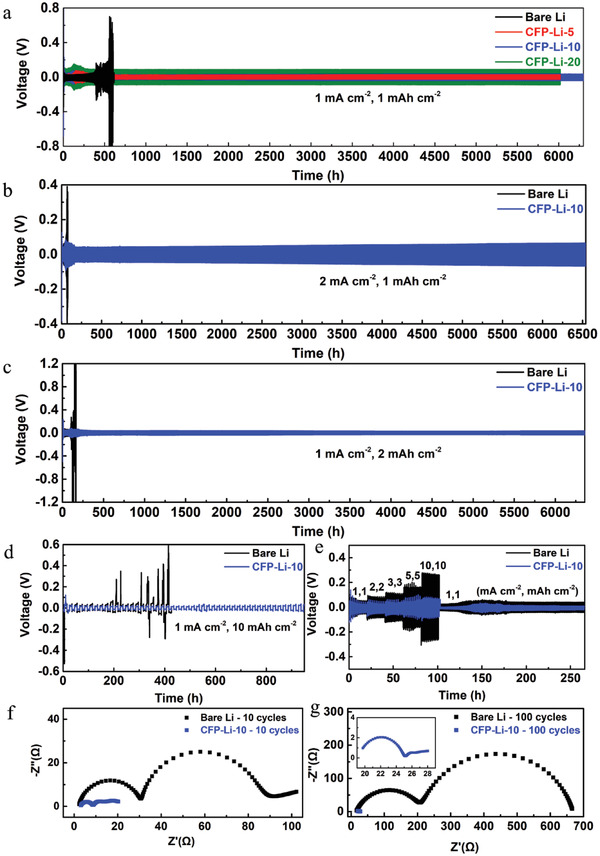
a) Electrochemical performance of symmetric cells containing bare Li, CFP‐Li‐5, CFP‐Li‐10, or CFP‐Li‐20 at current densities of 1 mA cm^−2^ and capacities of 1 mAh cm^−2^. b) Performance of bare Li and CFP‐Li‐10 cells at 2 mA cm^−2^ and 1 mAh cm^−2^, c) at 1 mA cm^−2^ and 2 mAh cm^−2^, and d) at 1 mA cm^−2^ and 10 mAh cm^−2^. e) Rate performance for bare Li and CFP‐Li‐10 cells at current densities and capacities from 1 mA cm^−2^ and 1 mAh cm^−2^ to 10 mA cm^−2^ and 10 mAh cm^−2^, respectively. f) Electrochemical impedance spectra (EIS) of symmetric cells of bare Li and CFP‐Li‐10 at 1 mA cm^−2^ and 1 mAh cm^−2^ after ten cycles, and g) after 100 cycles (Inset shows an enlarged EIS trace for the CFP‐Li‐10 cell after 100 cycles).

To investigate the electrochemical plating/stripping behavior of plasma‐treated Li, symmetric cells of bare Li and CF_4_ plasma‐treated Li foils (CFP‐Li‐5, CFP‐Li‐10, CFP‐Li‐20) were assembled and tested at various current densities and areal capacities. As shown in **Figure** **2**a, at a current density of 1 mA cm^−2^ with an areal capacity of 1 mAh cm^−2^, the symmetric cell comprised of bare Li (Figure 2a) operated for less than 400 h, with a high overpotential of >60 mV. Specifically, the overpotential increased sharply to 1930 mV after 568 h. In contrast, the symmetric cells containing CFP‐Li‐5, CFP‐Li‐10, or CFP‐Li‐20 (Figure 2a) had much longer lifespans of 5960, 6310, and 6010 h, respectively. For the CFP‐Li‐10 cell in particular, the overpotential decreased from 130 to 50 mV during the first few cycles and remained stable at 50 mV for more than 6000 h. Notably, as the plasma treatment time was increased, the overpotential for the cells increased: from 44 mV for CFP‐Li‐5, to 50 mV for CFP‐Li‐10, and then to 174 mV for CFP‐Li‐20. This effect can be attributed to the longer plasma treatment time that caused the thickness of the modified LiF and Li_2_C_2_ layers to increase. When current density was increased to 2 mA cm^−2^ at the same areal capacity of 1 mAh cm^−2^ (Figure 2b), the CFP‐Li‐10 cell could be cycled stably for 6510 h with an overpotential of 82 mV; the bare Li cell could only be cycled for 72 h. When discharge capacity was increased to 2 mAh cm^−2^ at a current density of 1 mA cm^−2^ (Figure 2c), the symmetric cell of CFP‐Li‐10 exhibited an overpotential of 76 mV with a lifespan of 6350 h. Under the same conditions, the cell containing bare Li could only cycle for 120 h with an overpotential of 118 mV. By further increasing the discharge capacity to 10 mAh cm^−2^ (Figure 2d), a long cycle lifespan of 950 h was achieved by the CFP‐Li‐10 cell; the symmetric cell with bare Li failed after 200 h. Figure 2e shows the rate performance of CFP‐Li‐10 and bare Li containing symmetric cells as measured while simultaneously increasing the current density and areal capacity. As the current density and discharge depth was increased from 1 mA cm^−2^ and 1 mAh cm^−2^ to 10 mA cm^−2^ and 10 mAh cm^−2^, respectively, the overpotential of the CFP‐Li‐10 cell increased from 50 to 150 mV while that of the bare Li cell increased from 100 to 550 mV. With a fixed current density of 1 mA cm^−2^ and increasing depth of discharge capacity, the CFP‐Li‐10 cell also exhibited a significantly better rate performance than the bare Li cell (Figure S6c, Supporting Information).

The low polarization and long cycle life of the symmetric cells containing plasma treated Li are both due to the fast Li^+^ diffusion kinetics and dendrite‐free Li electrodeposition induced by the artificial LiF/Li_2_C_2_ layers.^[^ ^39^ ^]^ Electrochemical impedance spectroscopy (EIS) was conducted to investigate the Li^+^ diffusion kinetics for bare Li and CF_4_ plasma‐treated Li after different cycles. After ten cycles (Figure 2f) the interfacial charge transfer resistance (*R*_ct_ = 11.0 Ω) and SEI resistance (*R*_SEI_ = 3.9 Ω) of CFP‐Li‐10 were significantly lower than for bare Li (*R*_ct_ = 45.9 Ω, *R*_SEI_ = 28.9 Ω), based on the equivalent circuit model (Figure S7b, Supporting Information). Moreover, after 100 cycles (Figure 2g) the *R*_ct_ (457.1 Ω) and *R*_SEI_ (195.4 Ω) of bare Li increased rapidly, but CFP‐Li‐10 still maintained low values of *R*_ct_ = 0.7 Ω and *R*_SEI_ = 0.5 Ω. The sharp increase in the impedance of bare Li is attributable to the rapid growth and accumulation of Li dendrites, while the comparatively lower impedance of CFP‐Li‐10 might be an effect of the modified layer's role in suppressing electrolyte decomposition and other side reactions.^[^ ^29^ ^]^ Moreover, the lower impedance of CFP‐Li‐10 indicated that the modified layers effectively improved the Li^+^ diffusion kinetics of the plating and stripping process, which was consistent with the better rate performance in symmetric cells. As illustrated in the SEM images of bare Li (Figure S8a, Supporting Information), a few lithium dendrites grew on the surface of bare Li after the initial striping/plating. As the number of cycles increased, needle‐like Li accumulated to form rough, pulverized structures. Numerous cracks appeared on the surface as well, which could be easily unthreaded by dendrites (Figure S8b,c, Supporting Information). However, for CFP‐Li‐10, Li metal preferred horizontal growth during the repeated striping/plating process; this resulted in a smooth surface and a dendrite‐free structure (Figures S8d–f, Supporting Information). Notably, the interfacial charge transfer resistance and SEI resistance for CFP‐Li‐5 (7.3 and 14.0 Ω, respectively) and CFP‐Li‐20 (8.9 and 17.3 Ω, respectively) were lower than the values for bare Li but higher than for CFP‐Li‐10. Thus, it is speculated that an artificial LiF/Li_2_C_2_ layer that is neither too thick nor too thin is most beneficial for fast and stable Li striping/plating. A thick coating layer would make it more difficult for the electrolyte to penetrate the anode, thus causing an uneven distribution of the electrolyte.^[^ ^40^ ^]^ However, if the protected layer is too thin, it cannot effectively suppress Li dendrite growth.

**Figure 3:**
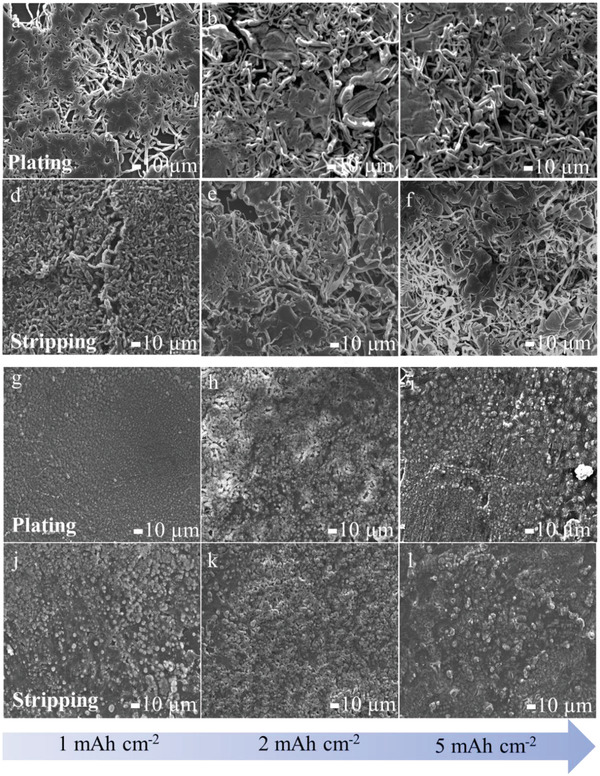
SEM images of Li plating/stripping morphologies on bare Li and CFP‐Li‐10 at a current density of 1 mA cm^−2^. a) Images after plating of bare Li at a capacity of 1 mAh cm^−2^, b) 2 mAh cm^−2^, and c) 5 mAh cm^−2^. d) Images after stripping of bare Li at 1 mAh cm^−2^, e) 2 mAh cm^−2^, and f) 5 mAh cm^−2^. g) Images after plating of CFP‐Li‐10 at 1 mAh cm^−2^, h) 2 mAh cm^−2^, and i) 5 mAh cm^−2^. j) Images after stripping of CFP‐Li‐10 at 1 mAh cm^−2^, k) 2 mAh cm^−2^, and l) 5 mAh cm^−2^.

To obtain a visual insight into the striping/plating process, the morphological evolution of lithium at various discharging states was observed by ex situ SEM and in situ optical microscopy (**Figure** **3**;Videos S1 and S2, Supporting Information). After plating at a capacity of 1 mAh cm^−2^ Li (Figure 3a), a small amount of pinpoint lithium dendrites appeared on the surface of the bare Li. Upon further increasing the plating capacity to 2 mAh cm^−2^ (Figure 3b), the dendrites grew longer and thicker, and numerous dendrites accumulated. When the capacity increased further to 5 mAh cm^−2^ (Figure 3c), Li dendrites covered the entire surface of Li and created a porous structure. The high surface area of Li dendrites typically increases the electrolyte consumption and lowers the coulombic efficiency of LMBs. In contrast, lithium was preferentially deposited inside the porous artificial layer for CFP‐Li‐10, with dense small nodules appearing on the surface as shown in SEM images (Figure 3g–i). When the plating capacity was increased from 1 to 5 mAh cm^−2^, nodule‐shaped Li structures formed and were uniformly distributed on the surface without the formation of Li dendrites. During the reversed Li stripping process (Figure 3j–l) these lithium nodules disappeared gradually, and the surface became smooth with negligible traces of dendrites. This indicated high reversibility of the stripping/plating process. However, for bare Li (Figure 3d–f), the dendrites stacked atop one another, cracked, and became unevenly distributed on the Li surface. The fragile dendrites could easily break off from the electrode and transform to be “dead” Li, leading to short circuits and poor cycling stability. Videos S1 and S2 (Supporting Information) show that, during the complete stripping/plating process, numerous lithium dendrites with thicknesses of order 500–600 µm appeared on bare Li. For the CFP‐Li‐10, however, the surface remained smooth and flat. These results verified that the modified layer composed of LiF and Li_2_C_2_ can induce uniform nucleation of the horizontal growth of lithium metal, thus inhibiting the formation and growth of lithium dendrites. In addition, the strong mechanical properties of the modified layer prevent the rupture of the SEI film during the platting/stripping process, thus guaranteeing the long‐term cycling stability and high safety of Li‐metal batteries.

**Figure 4:**
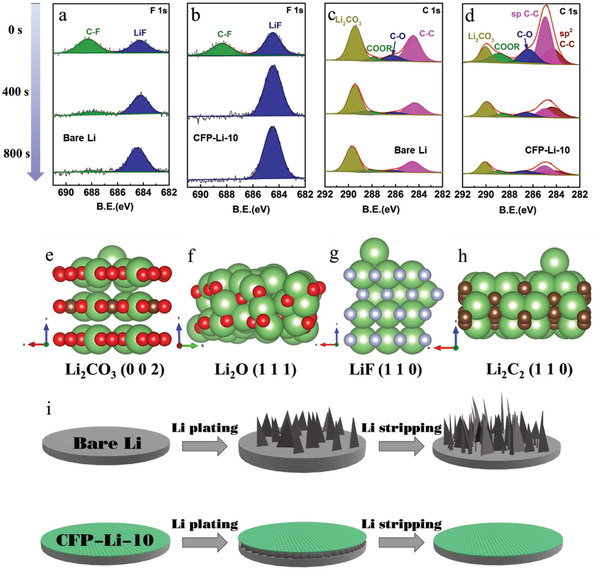
In‐depth XPS spectra for F 1s of a) bare Li and b) CFP‐Li‐10 after ten cycles. C 1s spectra for c) bare Li and d) CFP‐Li‐10 after ten cycles. Depiction of the crystal lattice plane and Li^+^ ions diffusion energy barriers for e) Li_2_CO_3_ (002), f) Li_2_O (111), g) LiF (110), and h) Li_2_C_2_ (110). i) Schematic diagram of dendrite inhibition by artificial LiF‐modified layers during Li plating/stripping.

In‐depth X‐ray photoelectron spectroscopy (XPS) characterization was also conducted to investigate the SEI composition after various amounts of cycling. In the high‐resolution F 1s spectra of both CFP‐Li‐10 and bare Li, the peaks located at 687.9 eV correspond to the C—F bond, while the peak located at a binding energy of 684.3 eV can be assigned to LiF (**Figure** **4**a,b). The LiF peaks for CFP‐Li‐10 are notably stronger than the C–F peaks, while in the spectra for bare Li the peaks associated with C‐F show higher intensity. In the high‐resolution C 1s spectra (Figure 4c,d), the fitted peaks located at 289.9, 288.8, 286.3, 285.0, and 284.3 eV can be assigned to the inorganic Li_2_CO_3_, organic COOR, C—O bonds, sp‐hybridized C, and sp^2^‐hybridized C, respectively. The amount of sp and sp^2^–hybridized C as well as organic C—O bonds were found to be higher in CFP‐Li‐10 than in bare Li, while the contents of inorganic Li_2_CO_3_ were lower (Figure 4c,d). These results demonstrate that the SEI layer of the CFP‐Li‐10 electrode after cycling was rich in LiF and Li_2_C_2_. Notably, an increased sputtering time caused a significant increase in the LiF peak for CFP‐Li‐10 and a gradual disappearance of the C‐F peaks. Further, the sp and sp^2^‐hybridized C and organic C—O bonds were reduced. It is conjectured that due to self‐healing of the SEI layers after repeated Li plating/stripping, LiF became concentrated in the inner part of the SEI while Li_2_C_2_ primarily existed in the outer layer.

To further analyze the role of LiF and Li_2_C_2_ in the improvement of Li striping/plating performance, DFT calculations were conducted to calculate the lithium‐ion adsorption energies and diffusion energy barriers for Li_2_CO_3_, Li_2_O, Li_2_C_2_, and LiF. Based on the experimental measurements, (002) crystal slabs of Li_2_CO_3_, (111) crystal slabs of Li_2_O, (110) crystal slabs of LiF, and (110) crystal slabs of Li_2_C_2_ were computationally built and optimized. The adsorption energy, *E*, was calculated according to the following equation:

(1) $$E=E_{sys}-E_{ion}-E_{slab}$$

where *E*_sys_, *E*_ion_, and *E*_slab_ represent the total energy after ion adsorption, the energy of Li ions, and the energy of the slab, respectively.

According to our calculation results, the adsorption energy of Li^+^ ions with LiF (−3.39 eV) and Li_2_C_2_ (−1.75 eV) is much higher than that with Li_2_CO_3_ (−0.25) and Li_2_O (−1.72 eV). The high lithium adsorption energy, especially for LiF, indicates outstanding lithiophilicity; this can ensure homogeneous nucleation and a smooth growth pattern for lithium, thus inhibiting dendrite growth and improving cycle stability. It was worth noting that the adsorption energy of Li ion on Li_2_CO_3_ (0 0 2) was −0.25 eV, which means that it was physical adsorption between them. In addition, the Li^+^ ion diffusion energy barriers (Figure 4e–h) for Li_2_O, Li_2_C_2_, and LiF were found to be 0.309, 0.097, and 0.143 eV, respectively. The physical adsorption effect makes the diffusion of lithium ion on the surface almost unlimited, so it is not practical to discuss the diffusion energy barrier of Li ions on the surface of Li_2_CO_3_.The Li‐ion diffusion energy barrier for Li_2_C_2_ was found to be the lowest, which implies that the materials is conducive to rapid ion transport on the Li_2_C_2_ surface; modification of Li_2_C_2_ could therefore accelerate the Li^+^ ion interfacial kinetics and reduce polarization in batteries.^[^ ^41^ ^]^ Combining the merits of LiF and Li_2_C_2_, the LiF–Li_2_C_2_ modified layers shown in this work induce uniform nucleation and horizontal growth of lithium, thus guaranteeing stable and dendrite‐free structures during the repeated plating/stripping process. In addition, the high mechanical strength of the artificial layers effectively restrains dendritic Li growth and helps accommodate the volumetric change of the electrode. These characteristics showcase the great potential in the application of plasma treated Li anodes in high‐performance lithium metal batteries that are stable over many cycles (Figure 4i).

**Figure 5:**
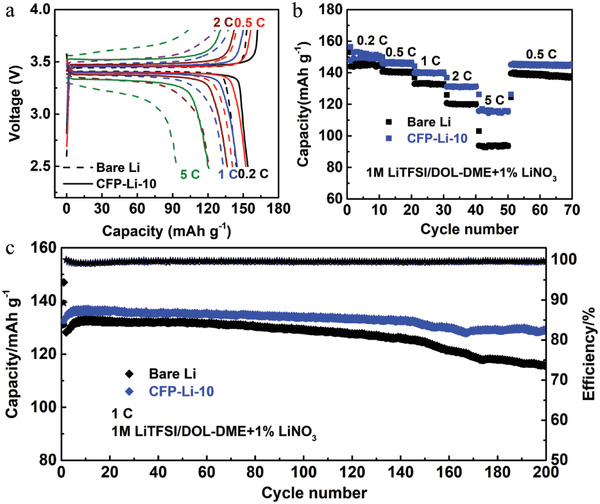
a) Discharge curves and b) rate performance of bare Li||LFP and CFP‐Li‐10||LFP full cells. c) Electrochemical performance of full cells containing bare Li or CFP‐Li‐10 at 1 C (at a current density of 170 mA g^−1^).

To evaluate the potential for practical application of plasma generated artificial LiF/Li_2_C_2_ layers on Li metal anodes, full cells of CFP‐Li‐10||LiFePO_4_ and bare Li||LiFePO_4_ were assembled with a high LiFePO_4_ (LFP) loading of 2.5 mg cm^−2^. As shown in **Figure** **5**c, the CFP‐Li‐10||LFP cell presented a relatively stable reversible capacity of ≈136 mAh g^−1^ at 1 C (at a current density of 170 mA g^−1^) with a capacity retention of 97.2% after 200 cycles. In contrast, the bare Li||LFP cell showed visible capacity decay over 200 cycles, with a capacity retention of only 87.9%. Moreover, the CFP‐Li‐10||LFP cell also demonstrated strong rate capability. As shown in Figure 5b, CFP‐Li‐10||LFP exhibited a reversible capacity of 149, 146, 140, 131, and 116 mAh g^−1^ over ten cycles at rates of 0.2 C, 0.5 C, 1 C, 2 C, or 5 C, respectively. Even after increasing the current density to 5 C, a high reversible capacity of 116 mAh g^−1^ was achieved with a voltage hysteresis of only 0.223 V (Figure 5a). In contrast, bare Li||LFP only delivered a capacity of 93 mAh g^−1^ with a higher voltage hysteresis of 0.424 V at 5 C. The stable cycling behavior, excellent rate performance, and low polarization can be ascribed to the enhanced ion transfer kinetics and dendrite‐free structure enabled by the LiF‐Li_2_C_2_ modified layer of CFP‐Li‐10.

## 3 Conclusion

In summary, a facile and rapid CF_4_ plasma treatment was conducted for the surface modification of a Li foil, and an artificial SEI layer composed of LiF and Li_2_C_2_ was fabricated for the first time. Benefiting from the high interfacial energy and mechanical strength of LiF, as well as the low Li^+^ diffusion barrier of Li_2_C_2_, the LiF‐Li_2_C_2_ composite layer enabled fast, stable plating and stripping of Li^+^ with dendrite‐free growth. CFP‐Li‐10||CFP‐Li‐10 symmetric cells exhibited excellent electrochemical performance, with a lifespan of up to 6500 h (with a current density of 2 mA cm^−2^ and capacity of 1 mAh cm^−2^). The CFP‐Li‐10||LFP full battery could operate stably for 200 cycles with negligible capacity decay. The rational design of the LiF–Li_2_C_2_ artificial layer thus provides a new perspective for the construction of high‐performance energy storage devices. Moreover, the room‐temperature plasma treatment described in this work is highly tunable as well as environmentally friendly, and can be easily applied in large‐scale energy storage applications.

## Conflict of Interest

The authors declare no conflict of interest.

## Supporting information

Supporting Information

Supplemental Video 1

Supplemental Video 2

## Data Availability

Research data are not shared.
