# Supplementary material for: CF4 Plasma‐Generated LiF‐Li2C2 Artificial Layers for Dendrite‐Free Lithium‐Metal Anodes
Source: Adv Sci (Weinh). 2022 May 26;9(21):2201147. doi: 10.1002/advs.202201147 (PMC9313480; doi:10.1002/advs.202201147)
Supplement: Supplementary file 1 — Supporting Information [file ADVS-9-2201147-s001.pdf]

Supporting information

CF_4 Plasma Generated LiF-Li_2C_2 Artificial Layers for Dendrite-Free Lithium Metal Anodes

Shengling Cao[a, b], *Xin He* [a], *Lanlan Nie* [a], *Jianwei Hu* [a], *Manlin Chen* [a], *Yu Han* [a], *Kangli Wang* [a], *Kai Jiang* [a], , *Min Zhou* [a] *

a State Key Laboratory of Advanced Electromagnetic Engineering and Technology, School of Electrical and Electronic Engineering, Huazhong University of Science and Technology, Wuhan, China

b State Key Laboratory of Materials Processing and Die & Mould Technology, School of Materials Science and Engineering, Huazhong University of Science and Technology, Wuhan, China

Corresponding author,

E-Mail address: minzhou0729@hust.edu.cn (M. Zhou)

1. Experimental Section

1.1. Fabrication of LiF/Li_2C_2 Decorated Li Foil (CFP-Li-5/10/20):

Commercially purchased Li foil (CEL corp. d=15.6 mm, thickness=60 μm) was placed into a stainless steel plasma chamber. The chamber was evacuated until the pressure reached 80 Pa, at which point CF_4 gas was allowed to flow into the chamber such that the pressure was maintained at 50 Pa for 5 min to remove the excess air in the chamber. A CF_4 plasma was then generated using a 13.56 MHz radio frequency emission source with a power of 200 W, and the Li foil treated for 5/10/20 minutes. The pressure in the chamber was maintained at 50 Pa during the reaction. The target product was obtained after cooling to room temperature (25℃), and named as CFP-Li-x (where x corresponds to the treatment time).

1.2. Material Characterization:

Grazing incidence XRD (GIXRD) was performed using a Rigaku Smart Lab diffractometer with a Cu Kα radiation (λ=0.1546 nm), and scanning electron microscopy (SEM) was performed using a JSM-7600F scanning electron microscope. X-ray photoelectron spectroscopy (XPS) was performed using a Thermo Scientific K-Alpha+ spectrometer with Al Kα radiation. Atomic force microscopy (AFM) was performed using a Bruker Dimension Icon instrument. *In situ* Optical Microscopy Videos were performed by LIB-MS-Ⅱ and LW750LJT metalloscope. Plasma emission spectra were obtained using HR+C1702 Ocean Optics.

1.3. Electrochemical Measurement:

Symmetric cells were assembled in an Ar glove box ($O_2 < 0.1$ppm, $H_2O < 0.1$ppm). CFP-Li-10 (d = 15.6 μm) was used as the electrode, and an untreated Li foil electrode was used as the comparison group. Celgard2400 (d = 19 μm) was used as the separator with an electrolyte of 40 μL 1M lithium bis(trifluoromethanesulfonyl) imide (LiTFSI) in a mixture of 1,3-dioxolane (DOL) and 1,2-dimethoxyethane (DME) (1:1 volume ratio) with the addition of 1 wt. % lithium nitrate (LiNO3). For the full cell, lithium iron phosphate (LFP), super P carbon, and polyvinylidene fluoride (PVDF) (8:1:1 weight ratio) were prepared in N-Methyl-2-pyrrolidone (NMP), coated on charcoal-coated aluminum foil, dried at 120 °C in a vacuum oven for 12 h, and cut into circular discs with a diameter of 12 mm. The active

material mass loading for the resulting electrodes was 2.5 or 4 mg cm^{-2}. CFP-Li-10||LFP and Li||LFP full cells were cycled between 2.5V and 3.8V at 1C. The symmetric cells and full cess were tested at a battery testing system (LAND Electronic Co., China). Electrochemical impedance spectroscopy (EIS) was performed for symmetric cells using CHI604E (Chenhua, Shanghai) electrochemistry workstation with frequencies ranging from 0.01 Hz to 10^6 Hz.

1.4. Theoretical Calculations:

Theoretical calculations were performed using the Vienna *ab initio* simulation package (VASP) code. The Electron core interactions is treated with the projector augmented wave (PAW) pseudopotential and Perdew−Burke−Ernzerhof (PBE) of the generalized gradient approximation (GGA) scheme is carried out for exchange-correlation potential. Considering the periodic structure, all slabs contain a 20 Å vacuum slab to avoid layer-to-layer interaction. The cutoff energy for the plane-wave basis set was set to 520 eV. The adsorption energy, E, was calculated according to the following equation:

$$E = E_{sys} - E_{ion} - E_{slab}$$

where E_{sys}, E_{ion}, and E_{slab} represent the total energy after ion adsorption, the energy of Li ions, and the energy of the slab, respectively. In this equation, the adsorption energy on the Li (1 1 0) slab is equal to the average atom energy in a bulk Li volume containing 16 Li atoms. Besides, because of the poor description of dispersion force in PBE functional, DFT-D3 is used to correct the dispersion force when calculating adsorption energy. Gaussian smearing was used for all calculations in this study. The smearing value, self-consistent field energy, and ionic force convergence tolerance were set to 0.1 eV, 1×10^{-6} eV, and 0.02 eV/ Å, respectively. The electron core interactions were treated with the projector augmented wave (PAW) pseudopotential, and the Perdew−Burke−Ernzerhof (PBE) of the generalized gradient approximation (GGA) scheme was carried out for the exchange-correlation potential. A 2 × 2 × 1 K-mesh was used for Brillouin zone sampling. The Climb image-nudged elastic band (CI-NEB) method using the LBFGS algorithm was used to calculate the diffusion energy barrier and ionic force convergence tolerance are set to 0.02 eV/ Å.

2. CF_4 Plasma Emission Spectrum

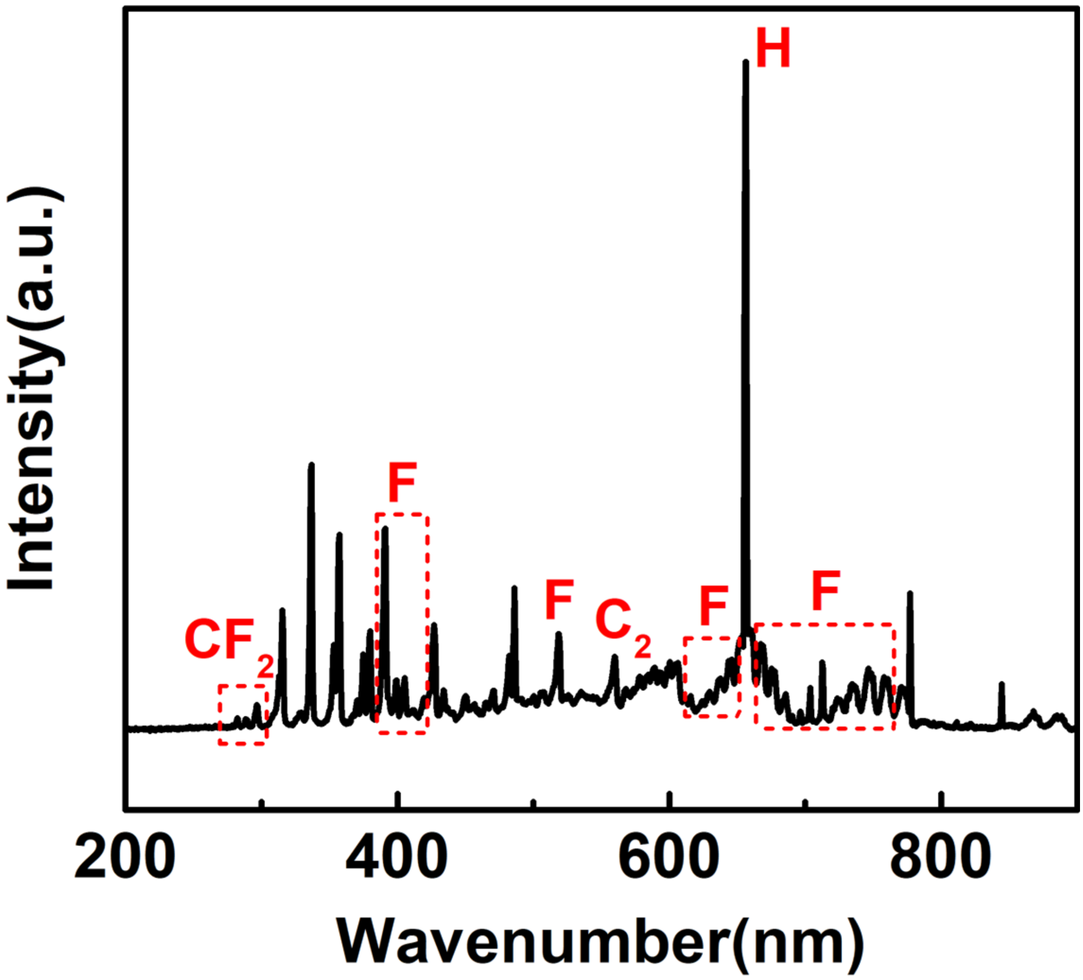

Figure S1. CF_4 plasma Emission Spectrum

3. SEM Images

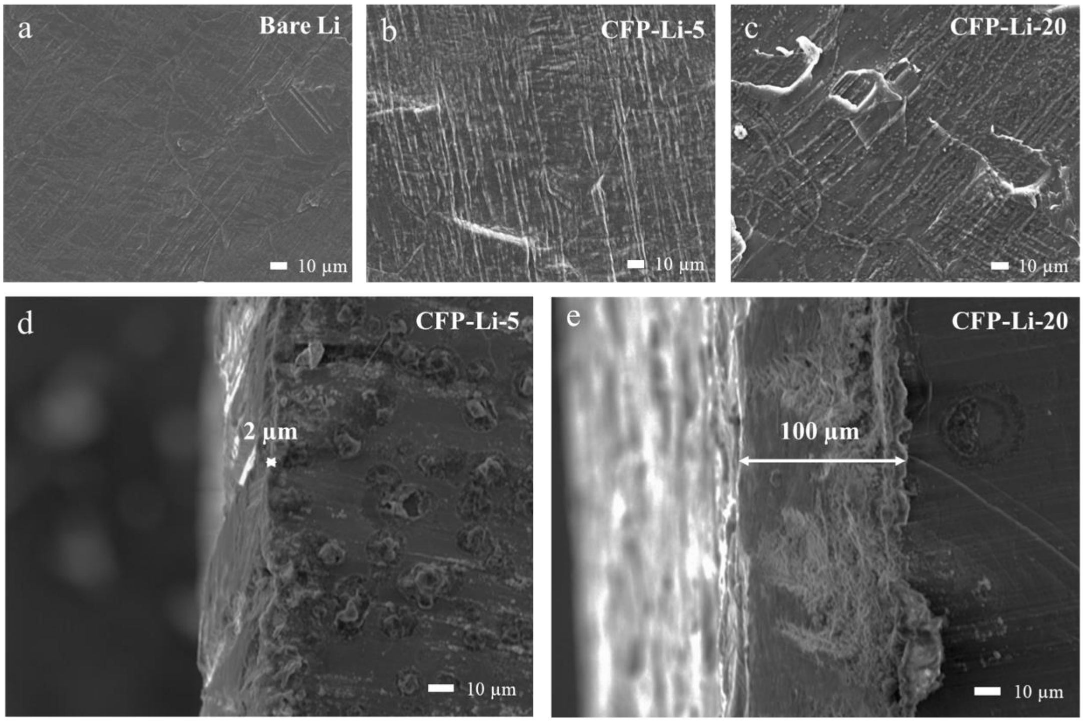

Figure S2. Surface SEM images of a) bare Li, b) CFP-Li-5, c) CFP-Li-20, and cross-sectional SEM images of d) CFP-Li-5, e)CFP-Li-20. The surfaces of the lithium foils treated with plasma for 5 and 20 min showed loose, streak-like structures, and the surface of the lithium foil treated for 20 min showed a small amount of breakage in the form of cracks.

4. AFM Topography and Young's Modulus Mapping

Surface morphology was characterized by AFM. Localized craters and raised structures were seen on the bare Li surface, and small, evenly distributed bulges were seen on the CFP-Li-10 surface. The surface roughness was compared between the two by the average surface root mean square (RMS). Bare Li showed an average RMS value of 79.1 nm, compared to 25.5 nm for CFP-Li-10. The lower RMS value indicates that CFP-Li-10 had a flatter and smoother surface, which is beneficial for improving the tip discharge effect and inhibiting lithium dendrite growth.

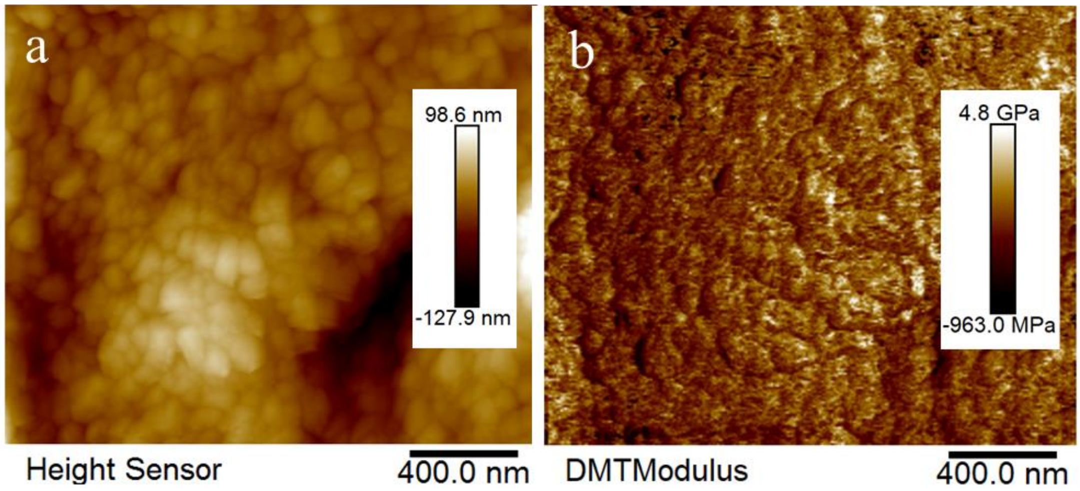

Figure S3. a) AFM topography and b) Young's modulus mapping of bare Li.

5. Electrolyte Contact Angle

The contact angle of electrolyte on CFP-Li-10 is 12.1°, the contact angle of electrolyte on bare Li is 17.3°, indicating the better wettability of the CFP-Li-10. The improved wettability of CFP-Li-10 accelerated the penetration of the electrolyte, thus facilitating rapid lithium-ion transport.

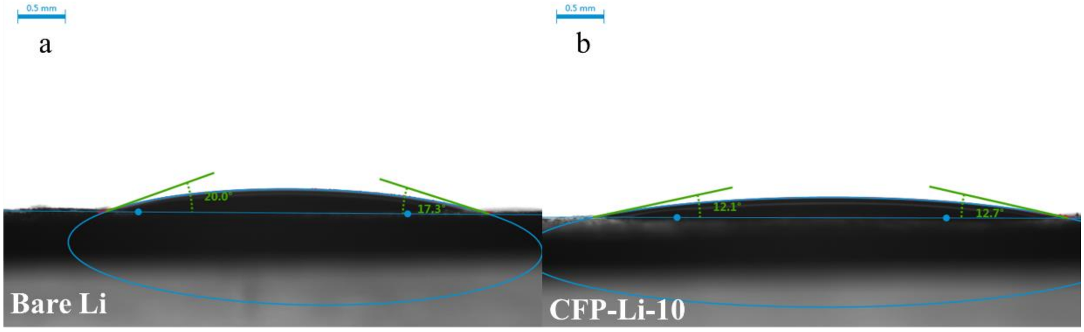

Figure S4. Electrolyte contact angle of a) Bare Li and b) CFP-Li-10.

6. SEM-EDS Line Scan Analysis

SEM-EDS line scan analysis exhibited an 18-μm-thick modified layer with a high carbon content in the outer layer and a high fluorine content in the inner layer, corresponding to the presence of Li2C2 and LiF, respectively. The high oxygen content in the outermost and innermost layers is associated with partial oxidation due to the lithium foils manufacturing and the testing process.

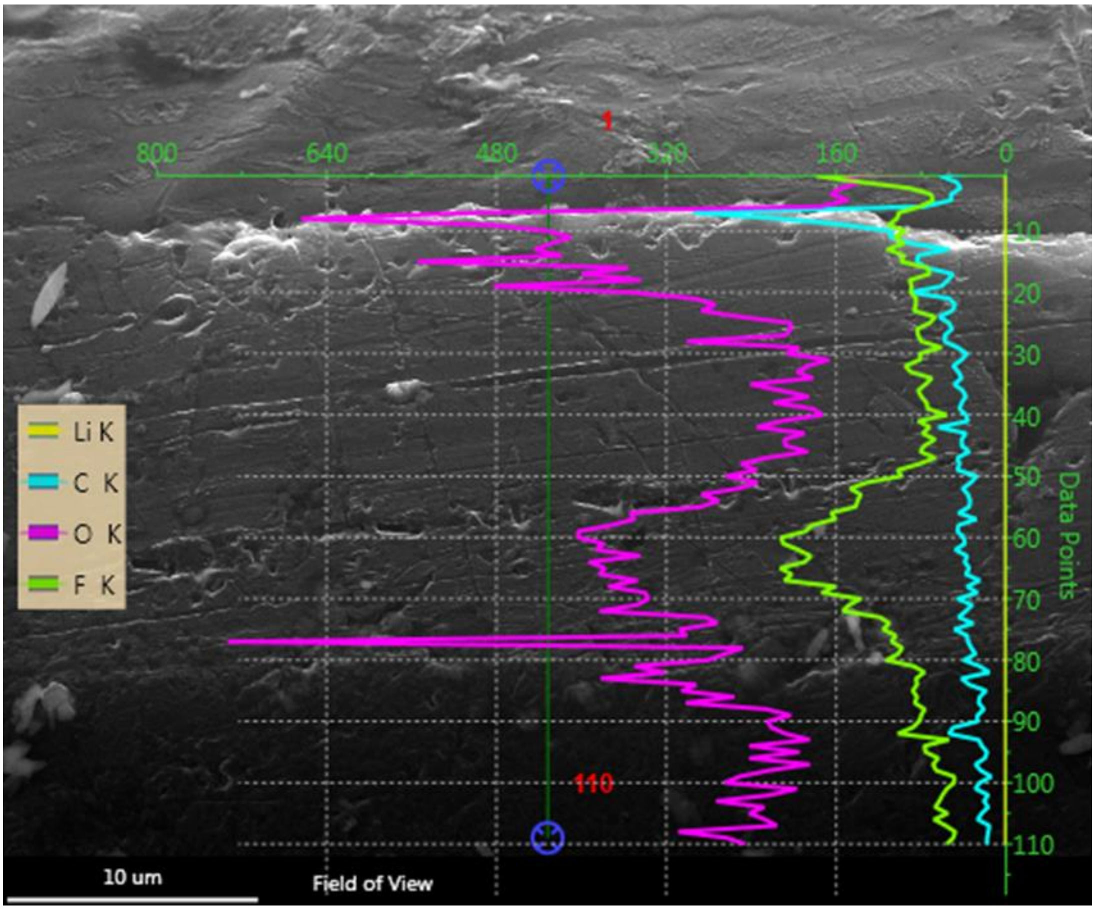

Figure S5. SEM-EDS line scan analysis of CFP-Li-10.

7. Electrochemical Performance

A parallel cells of bare Li and CFP-Li-10 as shown in Figure S7a. The symmetric cell comprised of bare Li operated for less than 550 h, with a high overpotential of >170 mV. For the CFP-Li-10, all three cells had much longer lifespans of more than 6000 h with a lower overpotential of 47, 47 and 76 mV, respectively. Cycled at a high current of 10 mA cm^{-2} and a high capacity of 10 mAh cm^{-2}, the bare Li symmetric cell exhibited an signigficantly high overpotential of 370 mV and failed after 205 h of cycling. The CFP-Li-10 cell, in contrast, exhibited an overpotential of only 114 mV and was cycled stably for 400 h.

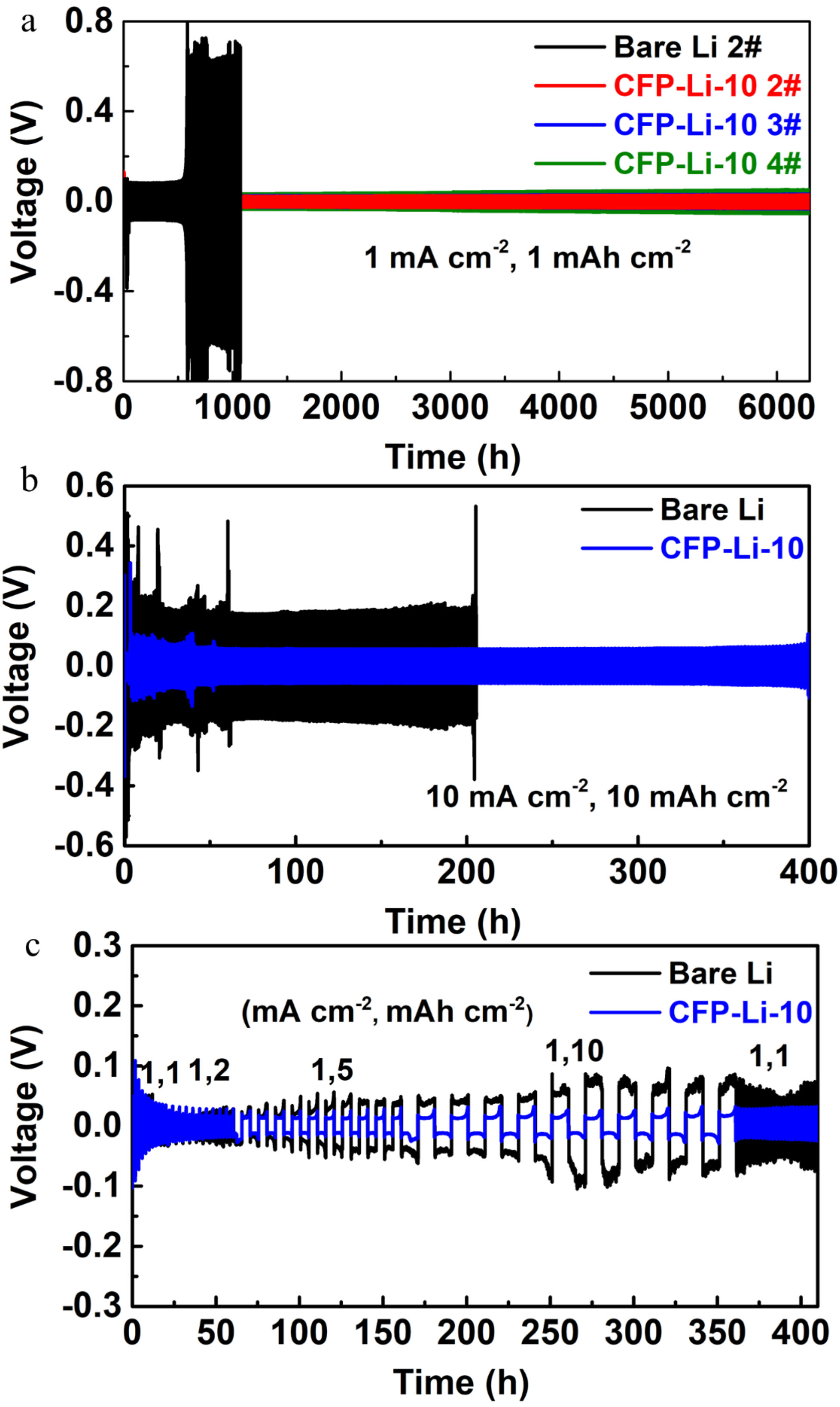

Figure S6. a) Electrochemical performances of symmetric cells of CFP-Li-10 and bare Li at a) 1 mA cm^{-2} and 1 mAh cm^{-2}, b) 10 mA cm^{-2} and 10 mAh cm^{-2}. c) Rate performance of CFP-Li-10 and bare Li.

8. **Electrochemical Impedance Spectra**

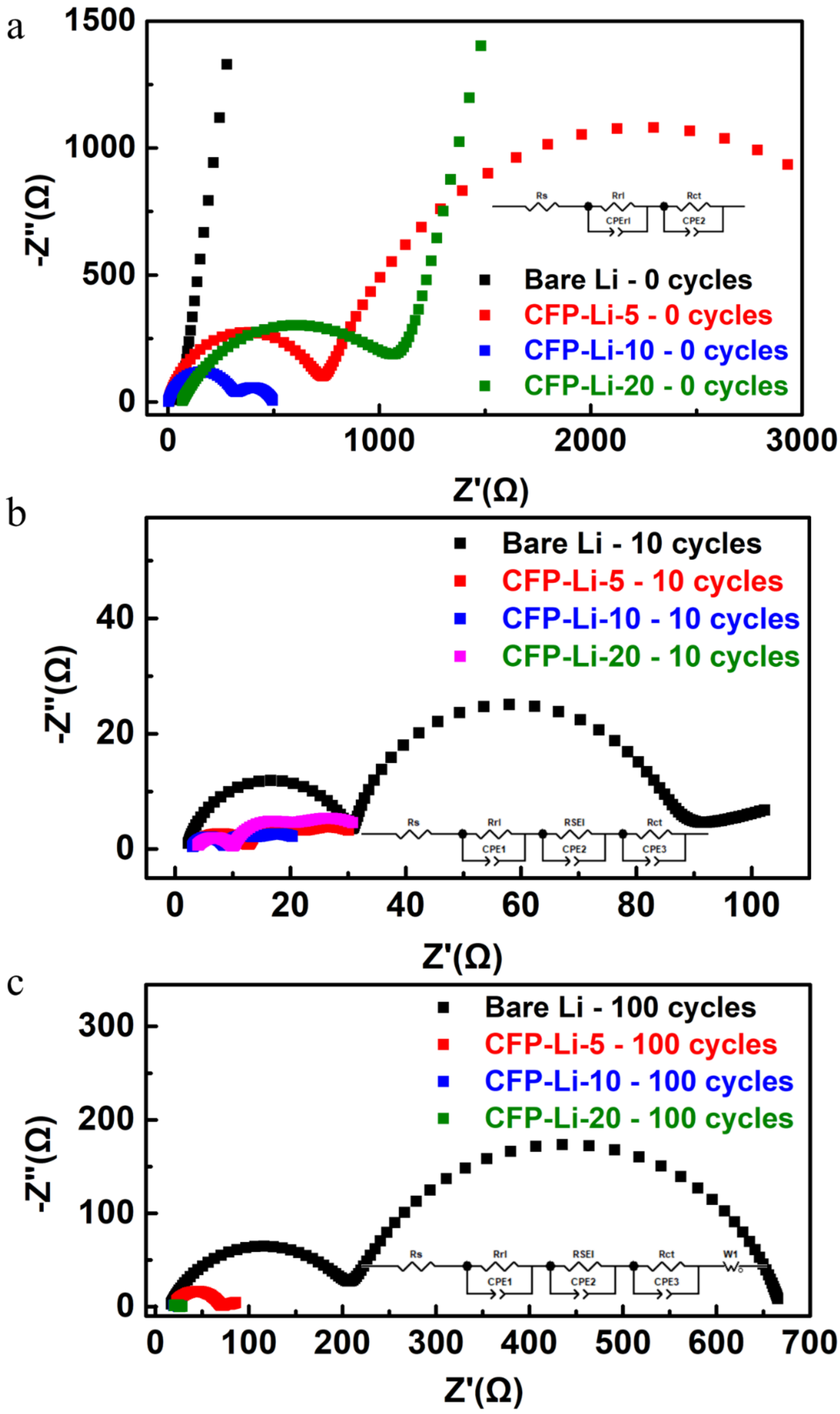

Figure S7. Electrochemical impedance spectra (EIS) of symmetric cells of bare Li, CFP-Li-5, CFP-Li-10 and CFP-Li-20 at 1 mA cm^{-2} and 1 mAh cm^{-2}: a) before cycling, b) after 10 cycles, and c) after 100 cycles.

To extract the values of each resistance component, different equivalent circuit models were constructed depending on the electrode system. The CFP-Li-10||CFP-Li-10 symmetric cell had a lithium-metal/LiF-Li_2C_2-modified layer, an SEI film, and an electrode/electrolyte interface. However, bare Li, lacked R_{rl}, which represents the reaction layer resistance in its equivalent circuit diagram, owing to the absence of LiF modification on the surface. R_s, R_{SEI}, and R_{ct} represent the solution resistance, SEI film resistance, and charge transfer resistance, respectively.

Table S1. Electrochemical impedance fitted data for symmetric cells of bare Li, CFP-Li-5, CFP-Li-10, and CFP-Li-20

0 cycle

Sample	R_{ct} [Ω]
Bare Li	56.7
CFP-Li-5	730.7
CFP-Li-10	186.8
CFP-Li-20	950.9

10 cycles

Sample	R_{SEI} [Ω]	R_{ct} [Ω]
Bare Li	28.9	45.9
CFP-Li-5	7.3	14.0
CFP-Li-10	3.9	11.0
CFP-Li-20	8.9	17.3

100 cycles

Sample	R_{SEI} [Ω]	R_{ct} [Ω]
Bare Li	185.4	457.1
CFP-Li-5	0.9	25.1
CFP-Li-10	0.5	0.7
CFP-Li-20	2.3	1.4

9. SEM Images

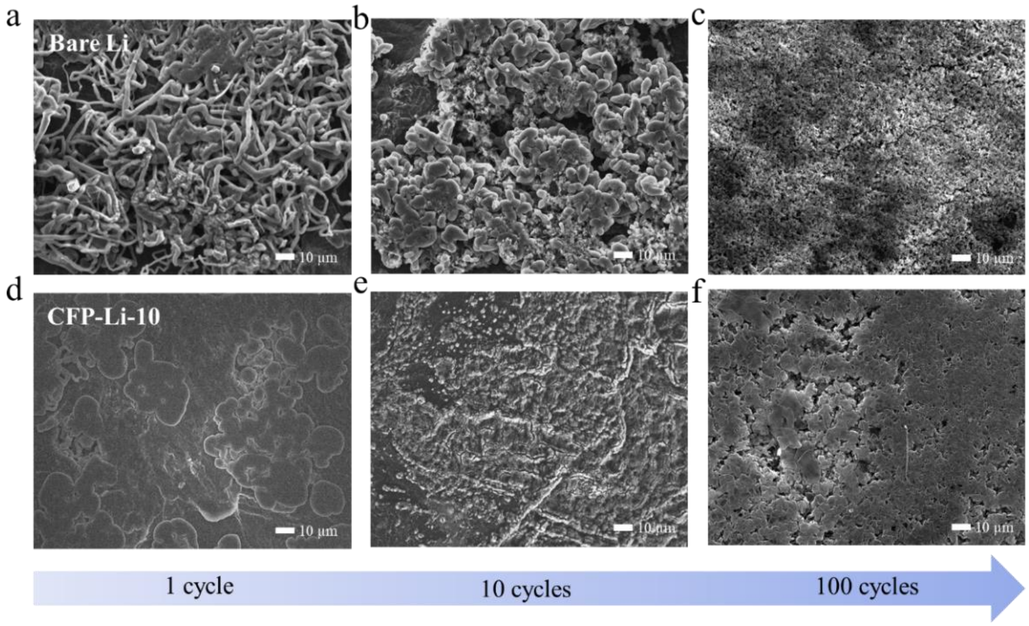

Figure S8. SEM images of bare Li after a) 1 cycle, b) 10 cycles, c) 100 cycles. SEM images of CFP-Li-10 after d) 1 cycle, e) 10 cycles, and f) 100 cycles.

10. Value of the Total Energy

Table S2. Value of the total energy after ion adsorption, the energy of Li ions, the energy of the slab, and the atom energy in a Li bulk

	E_{sys} [eV]	E_{ion} [eV]	E_{slab} [eV]
Li bulk	-31.557176		
Li_2C_2 (1 1 0)	-522.479908	-0.308730	-520.434876
LiF (1 0 0)	-610.914506	-0.308730	-609.893199
LiF (1 1 0)	-233.512205	-0.308730	-229.809958
Li_2CO_3 (0 0 2)	-474.109277	-0.308730	-473.550836
Li_2O (1 1 1)	-479.068067	-0.308730	-477.042976

All slabs contained at least four layers of atoms to ensure sufficient thinness. For the models select, the first step is to get the corresponding unit cell or supercell by DFT geometric optimization. On this basis, those cells are cleaved into corresponding surfaces and an additional vacuum slab is constructed. For Li, a 2×2×2 supercell was built and geometry optimization in 2×2×2 K-points. The optimized lattice constant of Li supercell is 6.88×6.88×6.88 Å (α=90°, β=90°, γ=90°). For LiF unit cell, geometry optimization is performed with K-points of 7×7×7. The optimized lattice constant of LiF unit cell is 2.53×2.53×2.53 Å (α=90°, β=90°, γ=90°). For Li_2C_2, the optimized lattice constant and K-points used for geometric optimization are 3.62×4.83×5.37 Å (α=90°, β=90°, γ=90°) and 5 ×3×3, respectively.

For slab geometric optimizations, the K-spacing value is 0.06 for generate K-Mesh. When the adsorption geometry is optimized, the bottom three layers are fixed, and only the adsorbed atom and the surface layer relax, so as to simulate the limited influence of adsorbed atom on the crystal structure in the actual situation as much as possible.

In addition, electron spin is considered in the calculation, especially the calculation of Li ion energy. In fact, the energy obtained regardless of spin is the difference between the total energy and the atomic energy defined in the pseudopotential file. Although It has no effect on the numerical trend, but it does have an effect on the energy accuracy.

11. The Diffusion Energy Barriers

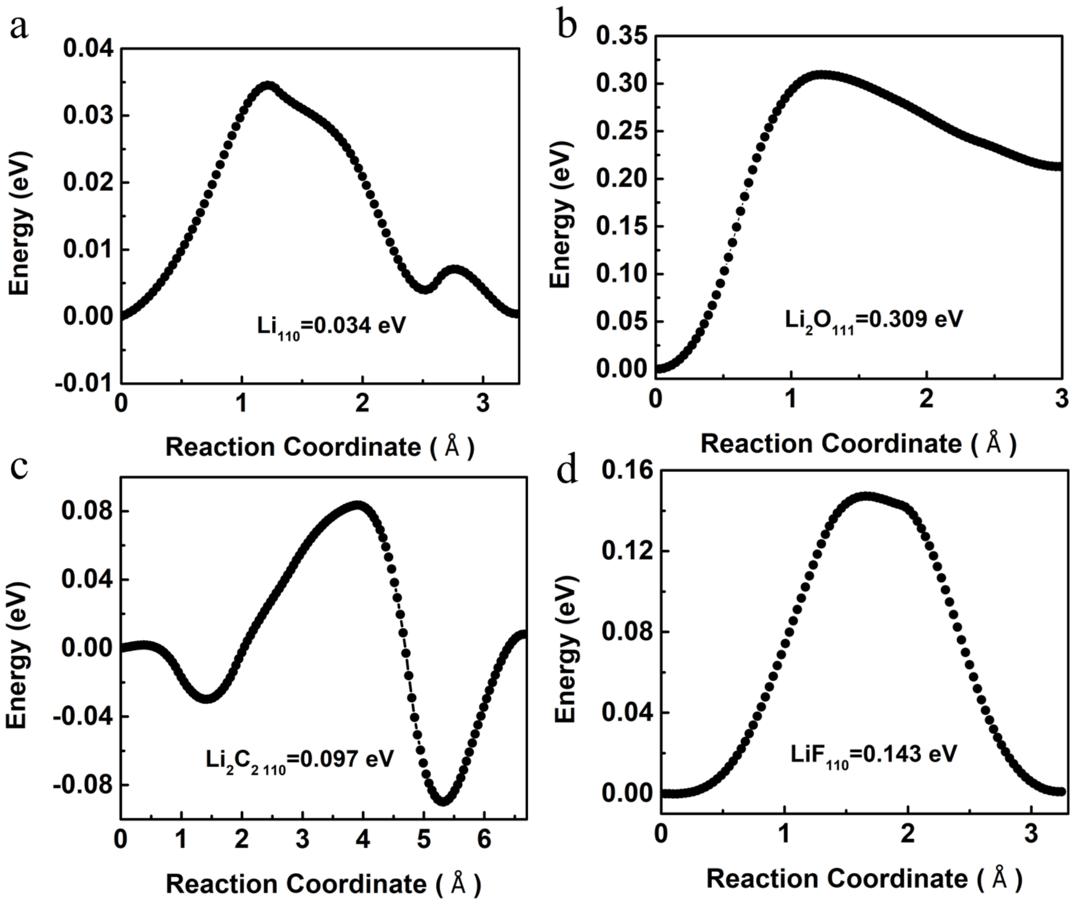

Figure S9. The diffusion energy barriers of Li atoms in a) Li (110), b) Li_2O (111), c) Li_2C_2 (110) and d) LiF (110).

12. XPS Spectra

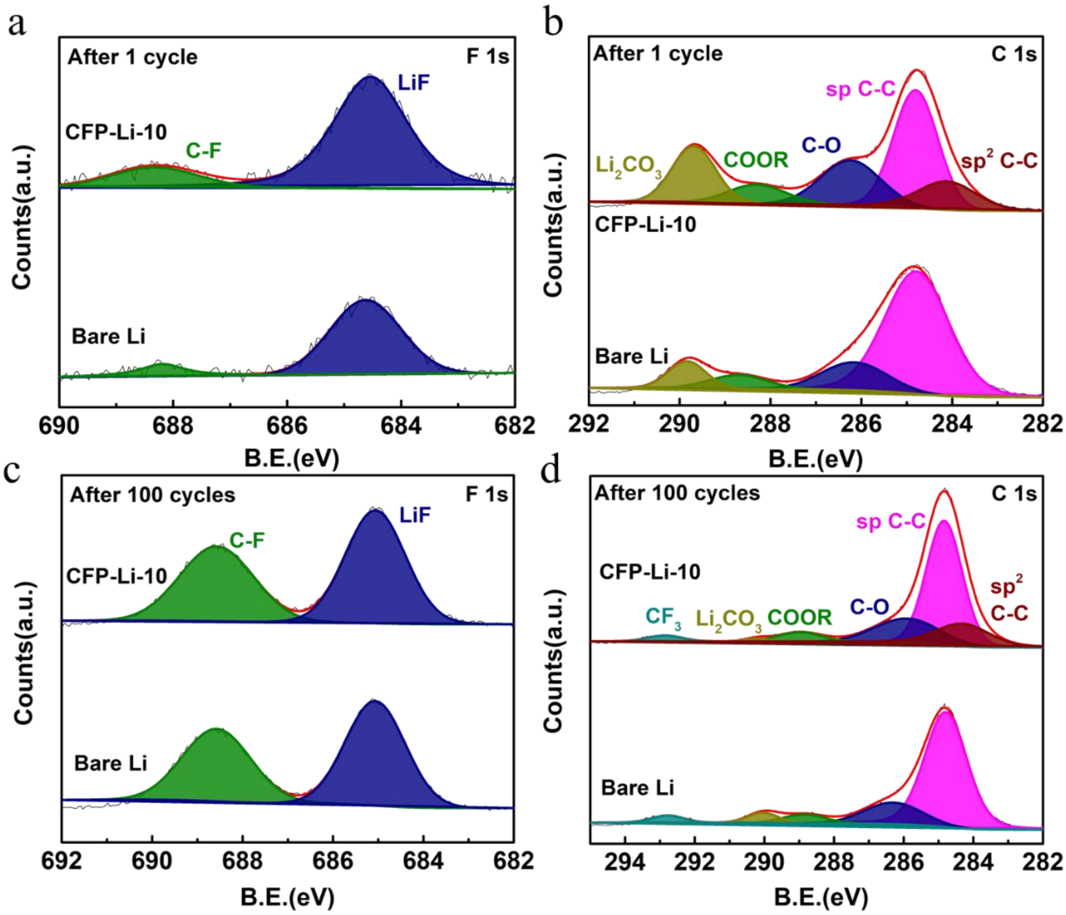

Figure S10. XPS spectra of CFP-Li-10 and bare Li after a, b) 1 cycle and c, d) 100 cycles.

13. Full Cells

Full cells with higher mass loading of LFP (4 mg cm^{-2}) have been assembled and tested (The amount of electrolyte of 80 μL). Full cells using CFP-Li-10 deliver a high reversible capacity of 156.5 mAh g^{-1} (at 1C) with considerable cycling stability (98.0% capacity retention over 170 cycles). In contrast, the bare Li||LFP cell showed visible capacity decay over 170 cycles, with a capacity retention of only 94.6%.

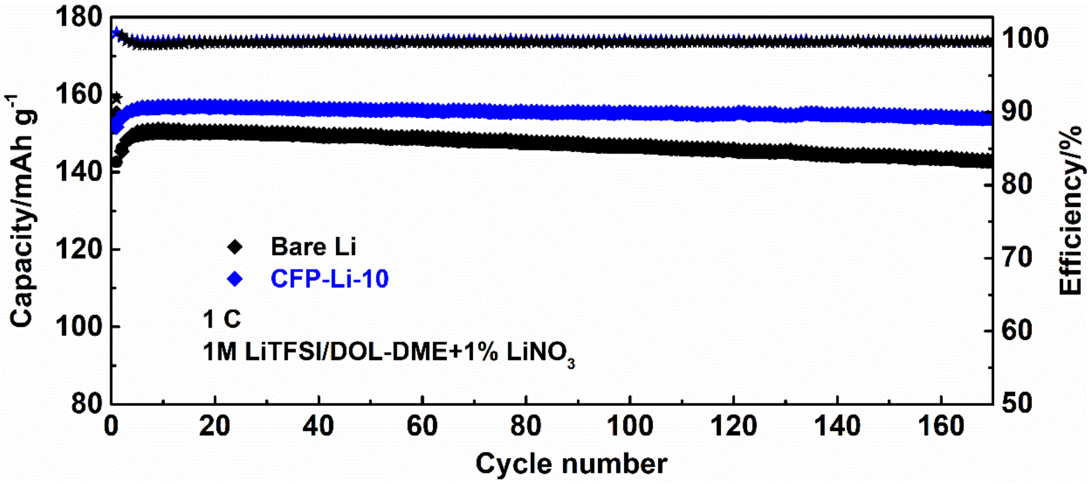

Figure S11 Electrochemical performance of full cells containing bare Li or CFP-Li-10 at 1 C (at a current density of 170 mA g^{-1}).

14. *In situ* Optical Microscopy Videos

bare Li.mp4

Video S1. *In situ* optical microscopy video of bare Li at 0.5 mA cm^{-2}.

CFP-Li-10.mp4

Video S2. *In situ* optical microscopy video of CFP-Li-10 at 0.5 mA cm^{-2}.
